# Health Care Access and Use Among Adults with Diabetes During the COVID-19 Pandemic — United States, February–March 2021

**DOI:** 10.15585/mmwr.mm7046a2

**Published:** 2021-11-19

**Authors:** Mark É. Czeisler, Catherine E. Barrett, Karen R. Siegel, Matthew D. Weaver, Charles A. Czeisler, Shantha M.W. Rajaratnam, Mark E. Howard, Kai McKeever Bullard

**Affiliations:** ^1^Turner Institute for Brain and Mental Health, Monash University, Melbourne, Australia; ^2^Austin Health, Melbourne, Australia; ^3^Harvard Medical School, Boston, Massachusetts; ^4^Division of Diabetes Translation, National Center for Chronic Disease Prevention and Health Promotion, CDC; ^5^Brigham and Women’s Hospital, Boston, Massachusetts; ^6^University of Melbourne, Melbourne, Australia.

Diabetes affects approximately one in 10 persons in the United States^†^ and is a risk factor for severe COVID-19 (*1*), especially when a patient’s diabetes is not well managed (*2*). The extent to which the COVID-19 pandemic has affected diabetes care and management, and whether this varies across age groups, is currently unknown. To evaluate access to and use of health care, as well as experiences, attitudes, and behaviors about COVID-19 prevention and vaccination, a nonprobability, Internet-based survey was administered to 5,261 U.S. adults aged ≥18 years during February–March 2021. Among respondents, 760 (14%) adults who reported having diabetes currently managed with medication were included in the analysis. Younger adults (aged 18–29 years) with diabetes were more likely to report having missed medical care during the past 3 months (87%; 79) than were those aged 30–59 years (63%; 372) or ≥60 years (26%; 309) (p<0.001). Overall, 44% of younger adults reported difficulty accessing diabetes medications. Younger adults with diabetes also reported lower intention to receive COVID-19 vaccination (66%) compared with adults aged ≥60 years^§^ (85%; p = 0.001). During the COVID-19 pandemic, efforts to enhance access to diabetes care for adults with diabetes and deliver public health messages emphasizing the importance of diabetes management and COVID-19 prevention, including vaccination, are warranted, especially in younger adults.

During February–March 2021, among 8,475 eligible U.S. adults, 5,261 (62.1%) completed the COVID-19 Outbreak Public Evaluation Initiative nonprobability, Internet-based survey administered by Qualtrics LLC.*^¶^* Respondents answered questions on demographic characteristics, attitudes and beliefs about COVID-19, and access to and use of medical care (including health care or telemedicine visits, delayed care, and loss of health insurance) since March 2020. The Human Research Ethics Committee of Monash University (Melbourne, Australia) reviewed and approved the study protocol on human participants research. This activity was also reviewed by CDC and was conducted consistent with applicable federal law and CDC policy.**

Among the 5,261 respondents, 760 (14%) who reported having diabetes currently managed by regular medications or treatment were included in the analyses.^††^ Demographic characteristics, experiences, attitudes, and behaviors related to the pandemic and health care access and use were assessed among these 760 persons. Demographic variables included age, sex, race/ethnicity, household income, education attainment, employment status, U.S. Census region,^§§^ urban/rural classification,^¶¶^ and health insurance status. Experiences, attitudes, and behaviors related to the pandemic included knowing someone who had received a positive test result for SARS-CoV-2 or who had died from COVID-19, perception of being at risk for severe COVID-19, vaccination intention, and composite measures of support for*** and adherence to recommended COVID-19 prevention behaviors^†††^ (e.g., wearing a mask, physical distancing, avoiding gatherings, and practicing hand hygiene). Regarding health care access and use, respondents reported whether they had delayed or avoided medical care because of concerns related to COVID-19,^§§§^ and whether their ability to access care or medications for diabetes was easier, harder, or unaffected as a consequence of the pandemic.

Weighted percentages and 95% CIs were calculated by age group (18–29, 30–59, and ≥60 years). CIs were calculated using a logit model. Significant differences (defined as p-values<0.05) among age groups were assessed using chi-square tests; statistical differences between groups were determined by nonoverlapping CIs only where chi-square tests were significant. Quota sampling and survey weighting were employed to match the U.S. Census Bureau’s 2019 American Community Survey population estimates for sex, age, and race/ethnicity of the general population. Analyses were conducted using the R survey package (version 3.29) and R software (version 4.0.2; R Foundation).

By age group, respondent characteristics varied by income, education, employment status, U.S. Census region, urban/rural classification, health insurance status, and diagnosed mental health conditions (all p<0.05) (Table 1). Adults aged 18–29 years (younger adults) less commonly reported having health insurance (77%), compared with those aged 30–59 years (91%) and ≥60 years (97%; p<0.001). Diagnosed mental health conditions, including depression, anxiety, and posttraumatic stress disorder, were more commonly reported among younger adults (86%) and adults aged 30–59 years (64%) than among adults aged ≥60 years (32%) (p<0.001).

**TABLE 1:** Demographic characteristics of adults with self-reported diabetes, by age — COVID-19 Outbreak Public Evaluation Initiative Survey, United States, February–March 2021

Characteristic	Age group, yrs						p-value
	18–29 (n = 79)		30–59 (n = 372)		≥60 (n = 309)		
	Weighted no.	% (95% CI)*	Weighted no.	% (95% CI)	Weighted no.	% (95% CI)	
**Sex**							
Male	45	57 (42–71)	224	60 (54–66)	180	58 (51–65)	0.941
Female	34	43 (29–58)	144	39 (33–44)	128	42 (34–49)	
**Mean age (95% CI), yrs**	23 (22–24)		45 (44–46)		70 (70–71)		
**Race/Ethnicity**							
White, non-Hispanic	31	40 (25–57)	211	57 (51–63)	168	55 (46.3–62.5)	0.144
Black, non-Hispanic	16	21 (13–32)	48	13 (9–18)	44	14 (9–21)	
Asian, non-Hispanic	6	8 (2–20)	18	—*	33	11 (6–17)	
Hispanic, any race	22	28 (17–43)	90	24 (19–31)	54	17 (10–28)	
**2019 household income, USD**							
<25,000	12	16 (9–27)	81	22 (17–28)	63	20 (14–29)	<0.001
25,000–49,999	37	48 (32–64)	51	14 (10–19)	75	24 (19–31)	
50,000–99,999	15	20 (11–33)	68	18 (14–24)	101	33 (25–41)	
≥100,000	10	—	158	42 (37–48)	58	19 (13–26)	
**Education**							
High school diploma or less	33	41 (26–58)	71	19 (14–25)	42	14 (9–19)	<0.001
College or some college	36	46 (31–62)	193	52 (46–58)	212	69 (61–75)	
After bachelor's degree	10	—	108	29 (24–34)	55	18 (13–25)	
**Employed**	55	70 (5–24)	258	70 (24–34)	35	11 (13–25)	<0.001
**U.S. Census region^†^**							
Northeast	8	—	93	25 (20–31)	38	12 (8–18)	0.006
Midwest	24	30 (18–47)	68	18 (14–24)	57	18 (13–25)	
South	39	50 (34–66)	148	40 (34–46)	148	48 (40–56)	
West	8	—	63	17 (13–22)	66	22 (15–30)	
**Rural/Urban residence^§^**							
Rural	26	33 (17–52)	53	14 (11–19)	55	18 (12–25)	0.015
Urban	53	67 (49–81)	318	86 (81–89)	253	82 (75–88)	
**Health insurance status**							
Yes	61	77 (60–89)	338	91 (85–94)	299	97 (93–98)	<0.001
No	13	—	33	9 (5–14)	4	—	
**Medical conditions** ^¶^							
Mental health	67	86 (67–96)	236	64 (57–69)	100	32 (25–41)	<0.001
Cardiovascular	61	77 (60–88)	277	75 (69–80)	256	83 (75–89)	0.190
Other	53	67 (48–83)	191	51 (45–58)	154	50 (11–25)	0.172

**Abbreviation:** USD = U.S. dollars.

* Data are weighted percentages, rounded to the nearest whole number. Rounded counts might not sum to expected values. Dashes represent percentages that are suppressed because relative SE>30%.

^†^ Region classification was determined by using the U.S. Census Bureau’s Census Regions and Divisions. https://www2.census.gov/geo/pdfs/maps-data/maps/reference/us_regdiv.pdf

^§^ Rural-urban classification was determined by using self-reported zip codes according to the Federal Office of Rural Health Policy definition of rurality. https://www.hrsa.gov/rural-health/about-us/definition/datafiles.html

^¶^ Selected underlying medical conditions included mental health (e.g., depression, anxiety, or posttraumatic stress disorder), cardiovascular (e.g., hypertension, cardiovascular disease, or high cholesterol), and other (e.g., any type of cancer or gastrointestinal disorder). Conditions were assessed using the question, “Have you ever been diagnosed with any of the following conditions?” with the response options: 1) “Never”; 2) “Yes, I have in the past, but don’t have it now”; 3) “Yes I have, but I do not regularly take medications or receive treatment”; and 4) “Yes I have, and I am regularly taking medications or receiving treatment.” Respondents who answered that they have received a diagnosis and chose either response 3 or 4 were considered to have the specified medical condition.

A larger proportion of younger adults with diabetes reported not knowing someone who had received a positive SARS-CoV-2 test result (90%) than did adults aged 30–59 years (69%) or ≥60 years (57%) (p<0.001) (Table 2). Both groups of adults aged <60 years were more likely to believe they were not at high risk for severe COVID-19 (94% [18–29 years], 76% [30–59 years]) than were adults aged ≥60 years (52%) (p<0.001). Younger adults reported the lowest support for COVID-19 prevention guidelines (28%) and COVID-19 prevention behaviors (30%), compared with adults aged 30–59 years (62% and 64%, respectively; p<0.001) and ≥60 years (51% and 72%, respectively; p<0.001). A lower proportion of younger adults reported that they intended to be vaccinated (66%) than did those aged ≥60 years (85%) (p<0.001).

**TABLE 2:** COVID-19 experiences, attitudes, and behaviors among adults with self-reported diabetes, by age — COVID-19 Outbreak Public Evaluation Initiative Survey, United States, February–March 2021

Characteristic	Age group, yrs						p-value
	18–29 (n = 79)		30–59 (n = 372)		≥60 (n = 309)		
	Weighted no.	% (95% CI)*	Weighted no.	% (95% CI)	Weighted no.	% (95% CI)	
**Know someone with a positive SARS-CoV-2 test result^†^**							
Yes	8	—*	117	31 (26–37)	134	43 (35–52)	<0.001
No	70	90 (81–95)	255	69 (63–74)	175	57 (48–65)	
**Know someone who died from COVID-19**							
Yes	8	—	57	15 (11–20)	69	22 (16–30)	0.048
No	71	90 (79–96)	315	85 (80–89)	240	78 (70–84)	
**Believe to be at high risk for severe COVID-19**							
Yes	4	—	90	24 (19–30)	148	48 (40–56)	<0.001
No	74	94 (86–99)	282	76 (70–81)	161	52 (44–60)	
**Total COVID-19 Prevention Support Index^§^**							
High	22	28 (17–41)	229	62 (55–67)	158	51 (43–59)	<0.001
Medium	31	40 (25–56)	102	27 (22–33)	100	32 (25–40)	
Low	26	—	41	11 (8–15)	51	17 (12–23)	
**Total COVID-19 Prevention Behavior Index^¶^**							
High	24	30 (19–45)	236	64 (58–69)	223	72 (64–79)	<0.001
Medium	32	41 (26–58)	91	25 (20–30)	74	24 (17–32)	
Low	23	—	44	12 (9–16)	12	4 (2–6)	
**Would get vaccinated with COVID-19 vaccine**							
Yes	52	66 (50–79)	284	77 (71–81)	261	85 (79–89)	0.001
Not sure	6	—	49	13 (8–14)	30	10 (6–15)	
No	21	26 (4–15)	39	11 (9–18)	18	6 (3–9)	

* Data are weighted percentages, rounded to the nearest whole number. Rounded counts might not sum to expected values. Dashes represent percentages that are suppressed because relative SE>30%.

^†^ Respondents were asked to select the following statement, if applicable: “I know someone who has tested positive for COVID-19.”

^§^ A COVID-19 Prevention Support Index represents summed responses to questions on whether participants believed nonessential workers should stay home, believed persons should always keep ≥6 ft of physical distance, believed groups of 10 or more persons should not be allowed, or believed dining inside restaurants should not be allowed. Respondents reported whether they strongly disagreed, disagreed, neither agreed nor disagreed, agreed, or strongly agreed to each individual statement. Summed responses were three-way split into high, medium, and low categories.

^¶^ A COVID-19 Prevention Behavior Index represents summed responses to questions on whether participants kept ≥6 ft apart from others, avoided groups of 10 or more persons, wore cloth face covering when in public, and washed hands or used sanitizer after touching high-touch public surfaces. Respondents reported the frequency (i.e., never, rarely, sometimes, often, or always) of each behavior during the last week. Summed responses were three-way split into high, medium, and low categories.

Younger adults with diabetes reported having the lowest percentage of in-person health care appointments (53%), compared with those aged 30–59 years (76%) and ≥60 years (85%) (p<0.001) (Table 3). Both groups of adults aged <60 years were more likely to report delayed health care (87% [18–29 years], 63% [30–59 years]) than were adults aged ≥60 years (26%) (p<0.001). Approximately two thirds of adults aged 18–29 years (66%) and 30–59 years (69%) with diabetes reported that their access to diabetes care was unaffected, whereas 91% of older adults reported that their access to diabetes care was unaffected (p<0.001). Adults with diabetes aged <60 years were less likely to report unaffected access to diabetes medications (44% [18–29 years], 72% [30–59 years]), than were adults aged ≥60 years (96%) (p<0.001).

**TABLE 3:** Reported health care experiences, attitudes, and behaviors in adults with self-reported diabetes, by age — COVID-19 Outbreak Public Evaluation Initiative Survey, United States, February–March 2021

Characteristic	Age group, yrs						p-value
	18–29 (n = 79)		30–59 (n = 372)		≥60 (n = 309)		
	Weighted no.	% (95% CI)*	Weighted no.	% (95% CI)	Weighted no.	% (95% CI)	
**Health services received since Mar 2020**							
In-person^†^	41	53 (37–68)	281	76 (70–81)	262	85 (79–89)	<0.001
Telehealth^†^	32	40 (26–57)	192	52 (45–58)	158	51 (43–60)	0.416
**Disruption in health care because of COVID-19**							
**Delayed or avoided care because of COVID-19–related concerns^§^**							
Any	69	87 (78–93)	232	63 (56–68)	80	26 (20–33)	<0.001
Urgent or emergency	37	47 (32–63)	90	24 (19–30)	12	—*	<0.001
Routine medical care	37	47 (31–63)	183	49 (43–55)	75	24 (18–32)	<0.001
No	10	—	139	38 (32–44)	229	74 (67–80)	<0.001
**Affected ability to access care for diabetes^¶^**							
Harder to access	19	—	102	28 (24–34)	24	8 (4–13)	<0.001
Not harder to access	52	66 (55–86)	255	69 (66–76)	282	91 (87–96)	
**Affected ability to access medication for diabetes^¶^**							
Harder to access	35	44 (33–67)	95	26 (21–32)	10	—	<0.001
Not harder to access	35	44 (33–67)	269	72 (68–79)	297	96 (94–98)	
**Reasons for disruption**							
**Disruption of transportation to health care facility**	7	—	34	9 (6–13)	15	5 (2–13)	0.335
**Personal concerns about receiving health care**							
Health care system may be overwhelmed	22	28 (17–42)	124	33 (28–39)	53	17 (12–24)	0.001
Me spreading SARS-CoV-2 at health care facility	22	28 (17–42)	73	20 (15–25)	11	—	<0.001
Becoming infected with SARS-CoV-2 at the health care facility	34	44 (28–61)	114	31 (25–36)	85	27 (21–35)	0.151
Becoming infected and infecting my household	15	—	95	26 (21–31)	60	20 (14–27)	0.406
**Concerns about the cost of the medical care**	5	6 (3–13)	33	9 (6–13)	17	6 (3–9)	0.280

* Data are weighted percentages, rounded to the nearest whole number. Rounded counts might not sum to expected values. Dashes represent percentages that are suppressed because relative SE>30%.

^†^ Health services for physical health, mental health, or substance abuse.

^§^ Respondents reported disrupted care in the past 3 months.

¶ Respondents were asked, “Has the pandemic affected your ability to access care and medication for diabetes?”

Among all respondents with diabetes, 28%, 33%, and 17% of those aged 18–29 years, 30–59 years, ≥60 years, respectively, reported that their health care was disrupted because of personal concerns that the health care system might be overwhelmed (p = 0.001). The most common reason for disruption in care among younger adults was concern about becoming infected with SARS-CoV-2 (44%), which did not significantly differ from that of adults aged ≥30 years (31% [30–59 years], 27% [≥60 years]; p = 0.151). Concerns about the cost of medical care did not differ significantly across the three age groups.

## Discussion

In this convenience sample of adults with diabetes, nearly nine in 10 (87%) younger adults (aged 18–29 years) reported delayed receipt of health care. In a previous survey (June 2020), 45% of adults aged 18–24 years, irrespective of diabetes status, reported delayed care or avoided health care.^¶¶¶^ Younger adults with diabetes largely did not consider themselves at risk for severe COVID-19 and reported the lowest engagement in preventive behaviors. Younger adults might be unaware of their own risk for severe COVID-19. Significantly fewer younger adults with diabetes reported health insurance coverage compared with older adults; thus, health policy interventions that increase access to health insurance coverage among younger adults with diabetes might be warranted.

Routine diabetes management is essential to mitigating risk for adverse health outcomes and severe COVID-19 in these patients (*3*); however, the pandemic might have contributed to disruptions in diabetes management, worsening of glycemic control, and increasing rates of severe diabetic ketoacidosis (*4*–*7*). Approximately 60% of patients with newly diagnosed type 1 diabetes experienced diabetic ketoacidosis as their first sign or symptom during April–August 2020, roughly twice as many as during previous years, suggesting delays in care-seeking behavior and diagnosis among persons with diabetes (*4*). Significant reductions in testing for hemoglobin A1c, an indicator of average blood glucose levels over the previous 2–3 months, were reported in 2020 (*5*). Use of telemedicine (*8*) or continuous glucose monitoring (*9*) might help improve glycemic control during the COVID-19 pandemic. However, others have reported worsening of glucose control through telehealth (*10*) and lower satisfaction with telehealth visits among persons with diabetes (*6*). It is also possible that use of telehealth might have led to missed diagnosis of diabetes in cases in which patients sought treatment for symptoms that were less severe than diabetic ketoacidosis. Increased accessibility of in-person medical services and improved telehealth services might help to maintain required diabetes care.**** Health care providers can follow CDC guidance for maintaining safe operations.^††††^

Persons with diabetes reported higher general and diabetes-related stress during the pandemic, which was associated with negative impacts on disease management, difficulty accessing diabetes care, and not adhering to COVID-19 precautions (*6*,*7*). Persons with diabetes are at increased risk for mental health issues.^§§§§^ In the present study, mental health conditions were approximately 2.5 times as likely in adults with diabetes aged 18–29 years (86%) as in adults aged ≥60 years (32%). Future research that assesses the impact of COVID-19 on mental health among persons with diabetes could further inform public health strategies in this population.

The findings in this report are subject to at least five limitations. First, quota sampling and survey weighting might not have eliminated inherent biases in this Internet-based convenience sample; thus, results might not be generalizable to all U.S. adults, including those with diabetes. Second, determination of diabetes was through self-report, and to increase specificity for diabetes, only respondents who reported having diabetes managed with medication were included; therefore, the findings are not representative of all persons with diabetes. Prevalence of diabetes managed with medication in this sample might be higher than would be expected in the larger U.S. population, potentially reflecting a higher diabetes prevalence and survey completion among older adults. Third, this survey is cross-sectional and causality between measures cannot be inferred. Fourth, participants were asked about their behavior during the preceding year, and responses are subject to recall bias. Similarly, temporal changes in participants’ access to medical care and attitudes around COVID-19 prevention were not assessed before or throughout the COVID-19 pandemic. This survey was conducted before emergence of the highly contagious SARS-CoV-2 B.1.617.2 (Delta) variant in the United States.^¶¶¶¶^ It is possible that younger adults might know more people who received positive test results since the Delta variant became prevalent in the United States, resulting in changing attitudes and behaviors not captured here. Finally, the small sample of adults aged 18–29 years with diabetes led to unreliable estimates for some measures and precluded multivariable analyses.

Adherence to diabetes care, including receiving COVID-19 vaccination, is important for managing risk for severe COVID-19 among persons with diabetes, including younger adults.***** Health care providers should recommend COVID-19 vaccination to all eligible persons, especially those at increased risk for severe COVID-19. Maintenance of diabetes management and promotion of health care–seeking behavior are essential for lifetime diabetes care. Future studies that assess factors affecting access to and use of care during the pandemic, particularly among younger persons with diabetes, could help inform tailored prevention strategies.

**Summary**

**What is already known about this topic?**

Persons with diabetes are at high risk for severe COVID-19, and the COVID-19 pandemic has affected diabetes care and management in the United States.

**What is added by this report?**

Among adults with diabetes, those aged 18–29 years reported the most disruption in access to and use of medical care and the least engagement in prevention of COVID-19, including vaccination intent.

**What are the implications for public health practice?**

Efforts are warranted to enhance access to diabetes care during the COVID-19 pandemic, and to deliver public health messages emphasizing the importance of diabetes management and COVID-19 prevention, including vaccination, especially among younger adults with diabetes.

## Footnotes

†
https://www.cdc.gov/diabetes/data/statistics-report/index.html

§ On December 20, 2020, the Advisory Committee on Immunization Practices recommended that persons with high-risk medical conditions, including type 2 diabetes, should be offered a COVID-19 vaccine in Phase 1C (https://www.cdc.gov/mmwr/volumes/69/wr/mm695152e2.htm). CDC classified type 1 and type 2 diabetes at the same risk level for severe COVID-19 on March 29, 2021; however, many states had previously categorized both types at the same level.

¶ The COVID-19 Outbreak Public Evaluation Initiative surveys included in this analysis were administered by Qualtrics, LLC (https://www.qualtrics.com), a commercial survey company with a network of participant pools with varying recruitment methodologies that include digital advertisements and promotions, word-of-mouth and membership referrals, social networks, television and radio advertisements, and offline mail-based approaches.

** 45 C.F.R. part 46, 21 C.F.R. part 56; 42 U.S.C. Sect. 241(d); 5 U.S.C. Sect. 552a; 44 U.S.C. Sect. 3501 et seq.

†† Diabetes diagnosis was ascertained by responses to the following question: “Have you ever been diagnosed with any of the following conditions?” with the response options 1) “Never”; 2) “Yes, I have in the past, but don’t have it now”; 3) “Yes I have, but I do not regularly take medications or receive treatment”; and 4) “Yes I have, and I am regularly taking medications or receiving treatment.” Respondents who chose response 4 regarding diabetes were considered to have diabetes.

§§
https://www2.census.gov/geo/pdfs/maps-data/maps/reference/us_regdiv.pdf

¶¶ Rural-urban classification was determined using self-reported zip codes according to the Federal Office of Rural Health Policy definition of rurality. https://www.hrsa.gov/rural-health/about-us/definition/datafiles.html

*** A COVID-19 Prevention Support Index represents summed responses to questions on whether participants believed nonessential workers should stay home, believed persons should always keep ≥6 ft of physical distance, believed groups of 10 or more persons should not be allowed, or believed dining inside restaurants should not be allowed. Respondents reported whether they strongly disagreed, disagreed, neither agreed nor disagreed, agreed, or strongly agreed to each statement. Summed responses were three-way split into high, medium, and low categories.

††† A COVID-19 Prevention Behavior Index represents summed responses to questions on whether participants kept ≥6 ft apart from others, avoided groups of 10 or more persons, wore a cloth face covering when in public, and washed hands or used sanitizer after touching high-touch public surfaces. Respondents reported the frequency (never, rarely, sometimes, often, or always) of each behavior in the last week. Summed responses were three-way split into high, medium, and low categories.

§§§ Delayed or avoided medical care was determined by response to the question, “Have you delayed or avoided medical care because of concerns related to COVID-19?” Delay or avoidance was evaluated for emergency (e.g., care for immediate life-threatening conditions), urgent (e.g., care for immediate non–life-threatening conditions), and routine (e.g., annual checkups) medical care.

¶¶¶
https://www.cdc.gov/mmwr/volumes/69/wr/mm6936a4.htm

****
https://www.cdc.gov/coronavirus/2019-ncov/hcp/telehealth.html

††††
https://www.cdc.gov/coronavirus/2019-ncov/hcp/us-healthcare-facilities.html

§§§§
https://www.cdc.gov/diabetes/managing/mental-health.html

¶¶¶¶
https://www.cdc.gov/coronavirus/2019-ncov/variants/delta-variant.html

*****
https://www.cdc.gov/coronavirus/2019-ncov/need-extra-precautions/people-with-medical-conditions.html
